# Experimental Assessment of the Sensitiveness of an Electrochemical Oscillator towards Chemical Perturbations

**DOI:** 10.1371/journal.pone.0050145

**Published:** 2012-11-21

**Authors:** Graziela C. A. Ferreira, Bruno C. Batista, Hamilton Varela

**Affiliations:** 1 Institute of Chemistry of São Carlos, University of São Paulo, São Carlos, São Paulo, Brazil; 2 Ertl Center for Electrochemistry and Catalysis, Gwangju Institute of Science and Technology, Gwangju, South Korea; University of Pittsburgh, United States of America

## Abstract

In this study we address the problem of the response of a (electro)chemical oscillator towards chemical perturbations of different magnitudes. The chemical perturbation was achieved by addition of distinct amounts of trifluoromethanesulfonate (TFMSA), a rather stable and non-specifically adsorbing anion, and the system under investigation was the methanol electro-oxidation reaction under both stationary and oscillatory regimes. Increasing the anion concentration resulted in a decrease in the reaction rates of methanol oxidation and a general decrease in the parameter window where oscillations occurred. Furthermore, the addition of TFMSA was found to decrease the induction period and the total duration of oscillations. The mechanism underlying these observations was derived mathematically and revealed that inhibition in the methanol oxidation through blockage of active sites was found to further accelerate the intrinsic non-stationarity of the unperturbed system. Altogether, the presented results are among the few concerning the experimental assessment of the sensitiveness of an oscillator towards chemical perturbations. The universal nature of the complex chemical oscillator investigated here might be used for reference when studying the dynamics of other less accessible perturbed networks of (bio)chemical reactions.

## Introduction

Biological organization is characterized by the presence of rhythms (typically from about mili-seconds to years) at all levels [1]. A central property of most living systems consists of the fairly independence of their internal rhythms with respect to external and internal parameters. A well-recognized example is that of the temperature regulation observed in warm-blooded animals at the cost of considerable amounts of energy and the interplay of intricate mechanisms of control. The concentration of some decisive chemical species may also be regulated as in the example of the pH of blood and for glycolysis [2]. In order to maintain control over, usually many, variables, complex mechanisms of regulation exist which take advantage mostly of negative feedback loops, which have the property of keeping a stationary (or oscillatory) state stable in face of disturbances [3], [4].

The term robustness stands for the property of keeping functionalities or parameters within a narrow window of change when there is a disturbance in the environment or in the concentration of key chemical species. When discussing the robustness of a given organism it is very important to point out which properties are robust and toward which disturbances they are so. High levels of robustness are usually associated to a high degree of complexity in the reaction network [5] but the increase in the number of elements of a given set of reactions may bring some weaknesses to the whole system commonly displayed as catastrophic behavior in face of rare type perturbations [6]. In this way, there is always a balance between robustness and sensitiveness in biological networks that establishes an optimum relation between network structure and the frequency of rare events [7]. Although sensitiveness may be deleterious in the context of robust systems, living organisms have adapted in order to take advantage of the former for sensing purposes and during morphogenesis for example.

Some attempts have been successfully made in order to quantify robustness of a given system. Those may rely on experiments for which some relevant properties like concentration of chemicals or oscillatory frequencies are recorded as a function of feasible parameter changes and the behavior of those properties analyzed. It is also becoming routine to establish a model for a given biological network and, with the aid of tools imported from the field of nonlinear dynamics, to evaluate the occurrence of bifurcations and the extent of stability of a desired property on parameter plane maps [7], [8]. Finally, ingenious approaches based on quenching of oscillatory reactions have been proposed that make it possible to test models quantitatively and compare the results with experiments allowing a systematic search for better representations of the reaction [9], [10].

Biological structures display several relations among coupled networks and it is usually difficult to analyze one of them without the interference of others. Because of the chemical nature of those biological networks it is usually informative to study chemical systems that resemble some of its properties or functionalities [11]–[12], [13], [14]. The interplay among chemical, electrical and transport properties makes electrochemical systems fascinating to be employed in experimental/theoretical investigations of complex dynamics [15]–[16], [17]. As an illustrative example, our Group has recently investigated the role of temperature on the oscillatory dynamics of typical electrochemical oscillators. It was found that, for instance, during the oscillatory electro-oxidation of methanol [18] the frequency of oscillations would increase as temperature rises but, quite surprisingly, we observed temperature compensation in frequency for the electro-oxidation of comparable molecules, viz. formic acid and ethylene glycol [19], [20]. The reaction of those molecules can thus be thought as one of the simplest yet very interesting experimental systems. Of late, Kiss and co-workers [21]–[22], [23], [24] have discussed the role of temperature and some electrical properties on the oscillation features. For a NDR oscillator, which typically oscillates under potentiostatic control, the authors found theoretically and experimentally that the frequency of oscillations is directly related to the square root of the velocity of charge transfer [21]. On the other hand however, for HN-NDR oscillators in which the full activity of the catalyst is inhibited by a poisoning process it was found that the frequency was directly proportional to the square root of the velocity of poisoning [22]. The overall increase in the value of those constants when temperature is elevated was also suggested to explain the oscillatorArrhenius-like behavior. The electrode area and the velocity with which the electrode is rotated were also found to be important for describing the locus of Hopf bifurcations [23]. Also important in the context of this work was the finding that by careful modification on the time-scales associated with the velocity of change of potential by a suitable input in the capacitance of the electrode the authors were able to either induce oscillations death or birth [24], [25].

The examples cited above show how parameters such as temperature and electric properties impact the oscillatory dynamics. However, this is not the whole story. The chemical environment surrounding an electrochemical oscillator definitely plays a decisive role on its integrity or stability. Reaction rates in electrochemical systems may be particularly susceptible to the presence of species dissolved in the electrolyte. The impact exerted by dissolved species on the rate of electrochemical steps is non-trivial in a sense that it depends in its turn on additional parameters. The primary effect of anion adsorption is the blockage of surface sites [26], generally leading to an inhibition on overall reaction rates, but it may also influence specific steps in the reaction scheme. On the other hand, it has been shown that some adsorbed species may inhibit the production of poisoning species like adsorbed CO [27], or tune the selectivity among different pathways of reaction in favor of those producing more electrons [28]–[29], [30], [31]. Either way, it is undisputable that anion adsorption plays an important role in surface chemistry, and as so its influence on dynamical aspects of electrochemical systems should not go unnoticed. Although less studied than under regular regime, the effect of anion adsorption on the oscillatory dynamics has been reported. To mention some examples, Sazou and collaborators studied the effects caused by halide anions during the oscillatory corrosion of iron and found that the nature of the adsorbent would greatly affect the complexity of oscillations and the induction period [32]. Malkhandi *et. al* investigated the effect of inhibiting anions on the oscillatory oxidation of carbon monoxide with experiments and also simulations [33], [34]. Schell and co-workers have extensively investigated the effects that adsorbed anions would display on some selected electrocatalytic oscillatory reactions [35]–[36], [37], [38], [39].

The present contribution outcomes the response of an electrochemical oscillator with respect to chemical perturbations of distinct magnitudes and explore the robustness of the core oscillatory state as well as the sensitiveness of some other properties with special emphasis on the mechanism behind this dependence. The oscillatory electro-oxidation of methanol on polycrystalline platinum was selected for this experimental study mainly because methanol is one of the simplest C1 molecule, the whole system is very reproducible and its dynamics is relatively known [18], [40]–[41], [42], [43], [44], [45]. The chemical perturbation consisted of the addition of different amounts of the electrosorbing anion trifluoromethanesulfonate, henceforth referred to as TFMSA, and the system was investigated under regular and oscillatory regimes. As further clarified latter, the combined use of a non-specifically adsorbing anion and the relatively simple dynamics of our electrochemical oscillator allows at specifically isolating the blocking effect an adsorbing anion would cause on a surface oscillator.

## Materials and Methods

All experiments were performed at 25°C using a conventional electrochemical cell containing a reversible hydrogen reference electrode in the same electrolytic solution, a sheet of platinum as working electrode with real area of 0.8 cm^2^ and a large area platinum ring counter-electrode. All potentials referred here are quoted with respect to the hydrogen reference electrode. The electrolyte was always an aqueous solution with 0.1 molL^−1^ of HClO_4_ (Merck, 99,99%) and eventually 0.1 molL^−1^ of methanol (J.T. Baker, 99.9%) and different amounts of trifluormethanesulfonate (97%) were added to the electrolyte. The solution was purged with argon (White Martins, 6.0) after the cell was built up and during experiments the purging happened only in the atmosphere of the cell. For the voltammetric experiments, the electrode was cycled about 20 times between 0.05 and 1.5 V before a final curve was collected, in order to guarantee a reproducible profile. In the case of the oscillatory curves, a normalizing procedure was performed before the collection, which consisted of cycling the electrode between 0.05 and 1.5 V about 10 times and then polarizing it at 0.05 V for 1 min. After that a final galvanostatic curve was recorded at the selected value of current and when the collection was over argon was purged directly inside the solution in order to remove soluble intermediaries from the vicinities of the electrode and prepare the system for a new set of experiments.

## Results and Discussion

For the sake of clarity, the results have been divided in three parts. In the next section we discuss some general aspects of the electrochemical characterization under non-oscillatory regime. In section 3.2 the problem of the intrinsic non-stationarity of the non-perturbed time-series is discussed in connection with the impact of the chemical perturbation on the induction period and on the oscillating time. The effect of the addition of TFMSA on the oscillation waveform is detailed in section 3.3. Finally, the sensitiveness of our oscillator is discussed in section 3.4.

### Initial Characterization

**Figure 1:**
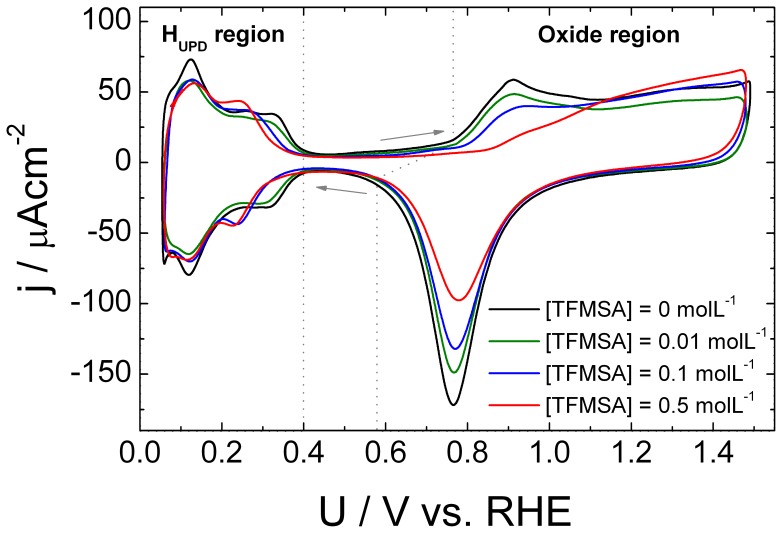
Cyclic voltammograms for different amounts of anion. Cyclic voltammograms for Pt(poly) in aqueous HClO4 0.1 molL^−1^ and in the presence of different amounts of TFMSA. Scan rate = 50 mVs^−1^, T = 25°C.

Figure 1 displays cyclic voltammograms registered at 50 mVs^−1^ exploring the electrochemistry of a polycrystalline platinum electrode immersed in a 0.1 M HClO_4_ aqueous solution containing different amounts of TFMSA. The major effects observed as the anion concentration increases are changes in the so called hydrogen UPD region between 0.05 V and 0.40 V as well as in the oxide region between 0.5 V and 1.5 V. It can be seen that the start of the hydrogen underpotential deposition during the reverse scan is shifted towards lower values of potential. Along the positive sweep, the end of the process of oxidation of adsorbed hydrogen also occurs at lower values of potential. Analyzing the positive scan it is seen that the anion causes a depletion in the peak at 0.12 V which corresponds to the oxidative desorption of weakly bonded UPD hydrogen. There is also a general decrease in the intensities of the peak located at 0.32 V and an increase for that observed at 0.24 V. However, the most profound effect observed is the decrease in the rates of production of platinum oxides. The beginning of the process is shifted towards higher values of potential and it can be seen that the amount of oxide produced, which is proportional to the area under the reduction peak, diminishes as the poison concentration increases. During the negative sweep the amount of oxide produced is decreased but the energies of those species seems to be barely affected since there is no displacement in the potential for reduction at 0.77 V. The H_UPD_ production display changes, which are mirrored by those found during the positive scan. The trends found for increasing amount of TFMSA mimic those found when the strength of adsorption of the anion is gradually increased from perchlorate to sulfate and finally in the presence of chloride anions [46].

These results are similar to those found during the study of UPD hydrogen under increasing influence of dissolved sulfate [47] and chloride anions [48]. Charge density-potential curves acquired for basal oriented platinum surfaces in perchloric media show that the potential of zero total charge is only slightly affected by the presence of 0.1 M TFMSA as expected for an anion that is known for not being specifically adsorbed [49]. It has been shown that the potential of zero total charge for a polycrystalline platinum electrode in aqueous perchloric acid media lies at about 0.3 V [50], so it is natural that the peak at 0.32 V be inhibited by anion adsorption. The appearance of the peak at 0.24 V can be related to the one found at 0.27 V for sulfuric acid media which is normally associated to the adsorption of hydrogen at Pt(100) sites [50].

**Figure 2:**
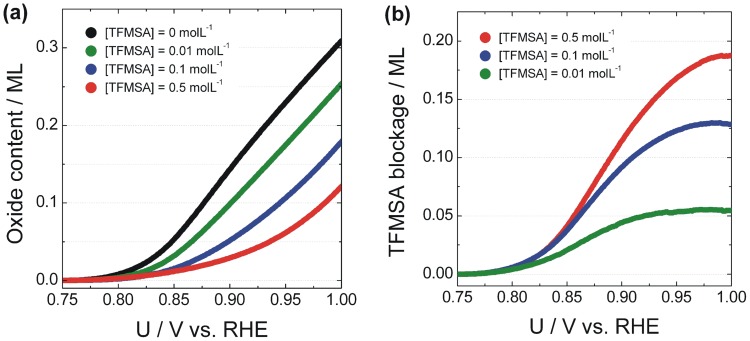
Coverage with oxides and anion as function of solution composition. (a) Coverage with platinum oxides as function of the applied potential for data presented in Figure 1. (b) Amount of platinum sites that were blocked by anion adsorption.

In order to quantify the inhibition caused by anion adsorption, it is illustrative to estimate the amount of oxide formed during the forward scan, *θ_ox_*, in units of monolayers (ML),
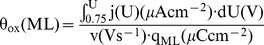
where *j(U)* represents the current density without the contribution of the double layer charging, *dU* is the integrand, ***v*** is the voltammetric sweep rate, and *q_ML_* is the charge of a PtO monolayer and amounts to 420 µCcm^−2^ [51], [52]. The extent of anion inhibition can thus be evaluated by subtracting the curve for platinum in the base electrolyte (i.e. aqueous 0.1 molL^−1^ HClO_4_) and that for a specific anion concentration. In other words, the reference condition is taken for the case where only perchlorate is present in the electrolyte. The result of those operations can be seen in Figure 2, which displays in panel (a) the amount of oxide produced, and in panel (b) the number of platinum sites blocked by anion adsorption.

**Figure 3:**
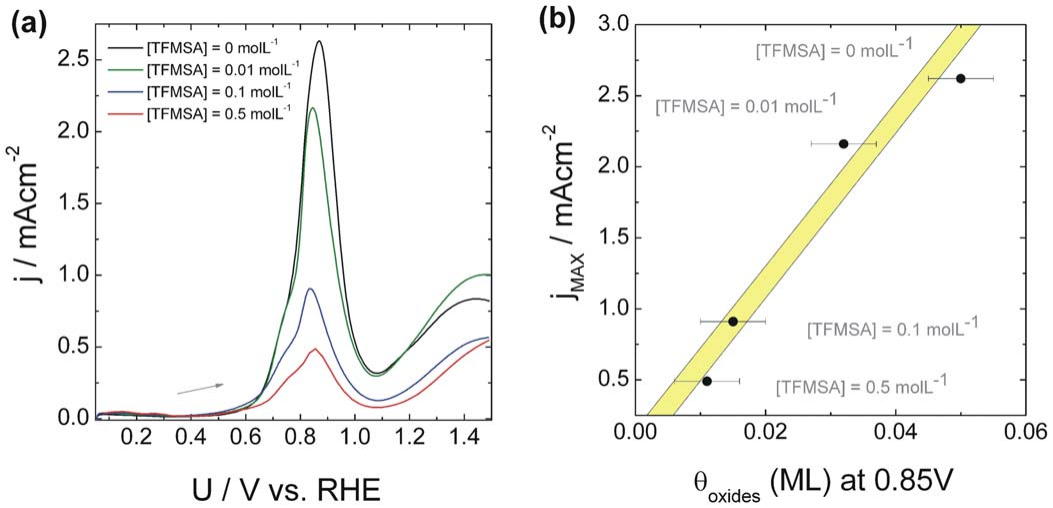
Cyclic voltammograms for methanol oxidation and relation with oxide content. (a) Cyclic voltammograms obtained for Pt(poly) for HClO_4_ 0.1 molL^−1^ and [MeOH] 0.1 molL^−1^ at a scan rate of 50 mVs^−1^. Several values for the concentration of TFMSA were used. (b) Total amount of oxides produced until 0.85 V (extracted from (a)) versus the maximum current found during methanol oxidation at distinct anion dosages. The error in evaluating the amount of oxides was estimated as ±0.005.

Panel (a) shows that there is a general decrease in the amount of oxide formed as the concentration of TFMSA increases. It has been argued that for potentials lower than 1.1 V water discharges at platinum directly producing oxides [53] and that this process is increasingly inhibited when anions with increasing strength of adsorption are present in the reactive medium [54]. The diminishment found in panel (a) can thus be associated with anion blockage and this phenomenon can be properly quantified by subtracting the curve for a given concentration of TFMSA from that for pure platinum as shown in panel (b). Figure 3 (a) presents the forward scan of a cyclic voltammogram obtained at 50 mVs^−1^ during the electrocatalytic oxidation of 0.1 molL^−1^ of methanol, and in the presence of different amounts of dissolved TFMSA. A pre-peak is observed at about 0.72 V, which is followed by a major oxidation peak at 0.85 V. After the electrode reaches the region of oxide production, the rates of reaction decrease dramatically and as the potential is elevated another wave of oxidation becomes apparent from 1.1 V on. It can be seen that the added TFMSA barely influences the position of the oxidation peak at 0.85 V, although its intensity is accordingly reduced as the amount of TFMSA increases.

The decrease in the oxidation currents observed is expected when an adsorbing species blocks surface sites for the reaction of the organic species. It is desirable to seek a correlation between the blockage of oxide production and the decrease in the rate of reaction for methanol. Figure 3 (b) displays the amount of oxides produced until the potential of 0.85 V is reached and the values of maximum current during methanol oxidation at the same potential. It can be seen that there is reasonable correlation between the normalized amount of oxides produced until 0.85 V and the maximum value of current observed. This result shows that besides glazing at the specific inhibition of the adsorption of methanol it is important to consider also that the anion postpones the initial stages of water oxidation towards higher potentials. Since the electro-oxidation of methanol critically depends on the amount of oxygenated species adsorbed at the surface, particularly through the steps involving production of formic acid and CO_2_, the influence of the anion on the production of adsorbed oxygenated must be regarded as important.

### Non-stationarity, Induction Period and Oscillating Time

**Figure 4:**
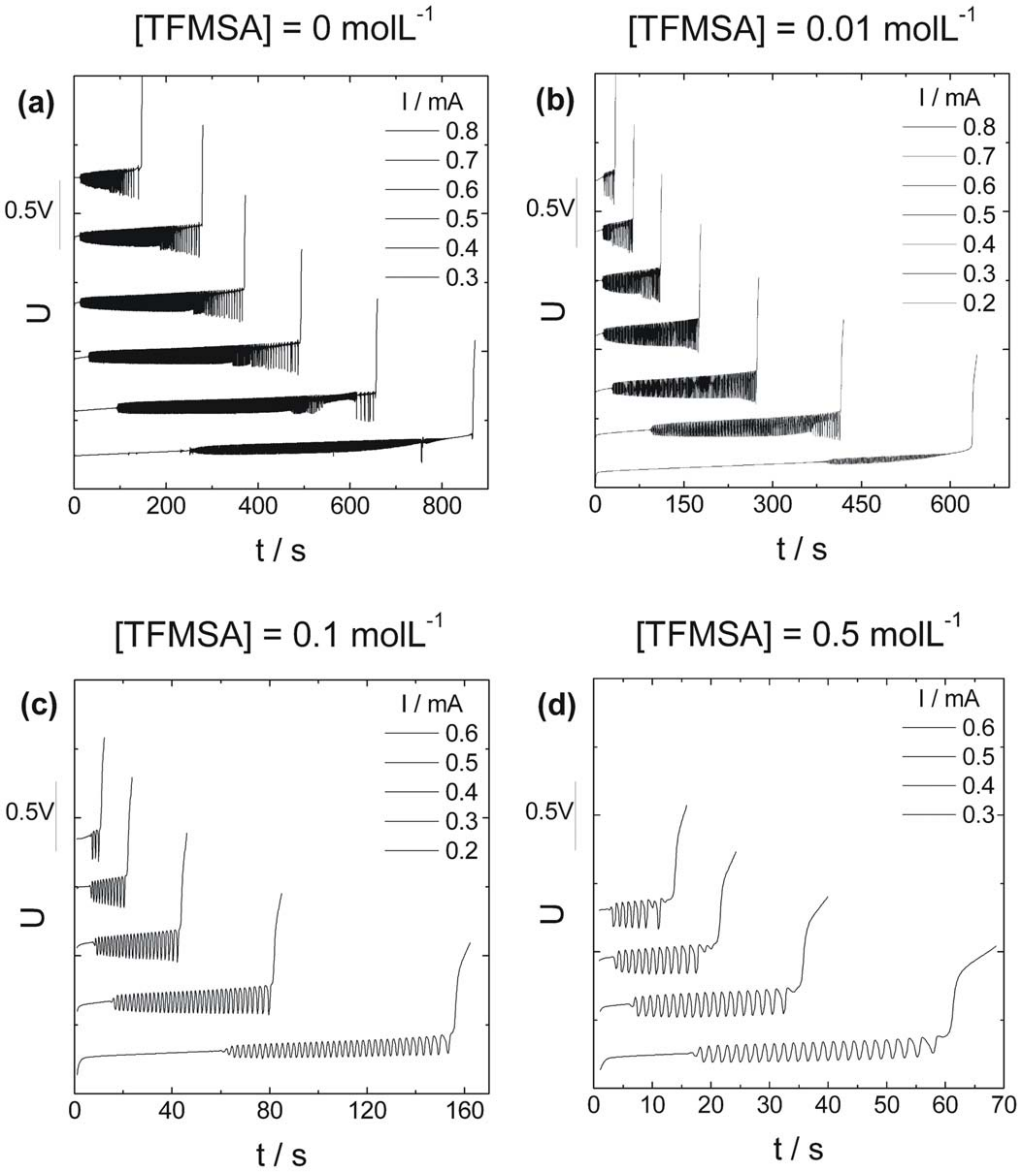
Oscillatory methanol oxidation in the presence of different amounts of anion. Galvanostatic curves obtained for Pt(poly) for [HClO_4_] = 0.1 molL^−1^ and [MeOH] = 0.1 molL^−1^. [TFMSA] was: a) 0 molL^−1^, b) 0.01 molL^−1^, c) 0.1 molL^−1^, d) 0.5 molL^−1^, and the applied currents ranged from values between 0.2 mA and 0.8 mA.

Figure 4 shows a compilation of the potential time-series registered at applied currents between 0.2 mA and 0.8 mA and in the presence of different amounts of TFMSA. A standard procedure was followed for the collection of each time-series, which consisted of initially applying 10 cycles of a cyclic voltammetry between 0.050 V and 1.5 V followed by the application of 50 mV for 1 min. Finally, the current was stepped to the desired value. This procedure was adapted from that used by Krausa and Vielstich [40] and ensured fairly reproducible results. The time-series usually fell in the same range of potentials but for clarity of presentation they were displaced in the y-axis.

In the absence of dissolved TFMSA it is generally seen that a given curve starts in a stationary way spending some time in the induction period. After that oscillations set in via a supercritical Hopf bifurcation giving birth to almost harmonic cycles. Those may eventually evolve into more complex ones displaying several small amplitude oscillations intercalated by a large amplitude excursion. The main observation that results from the addition of TFMSA is the prevalence of simpler oscillations and, more importantly, the decrease in the number of cycles.

For a given applied current, say 0.3 mA, as the concentration of anion increases there is an appreciable decrease in the induction period. Compare for example the situation for pure methanol, which starts oscillating at 250 s with that found for TFMSA 0.1 molL^−1^ which begins at 17 s. The ratio between those intervals is of about 15 and since the current is identical, the ratio between the oxidation charge produced is also 15. Such a great difference in charge and hence in the amount of products formed means that the accumulation of partially oxidized dissolved products is not fundamental to the birth of oscillations. The analysis that follows will shed light on what parameter is common to the beginning of oscillations.

**Figure 5:**
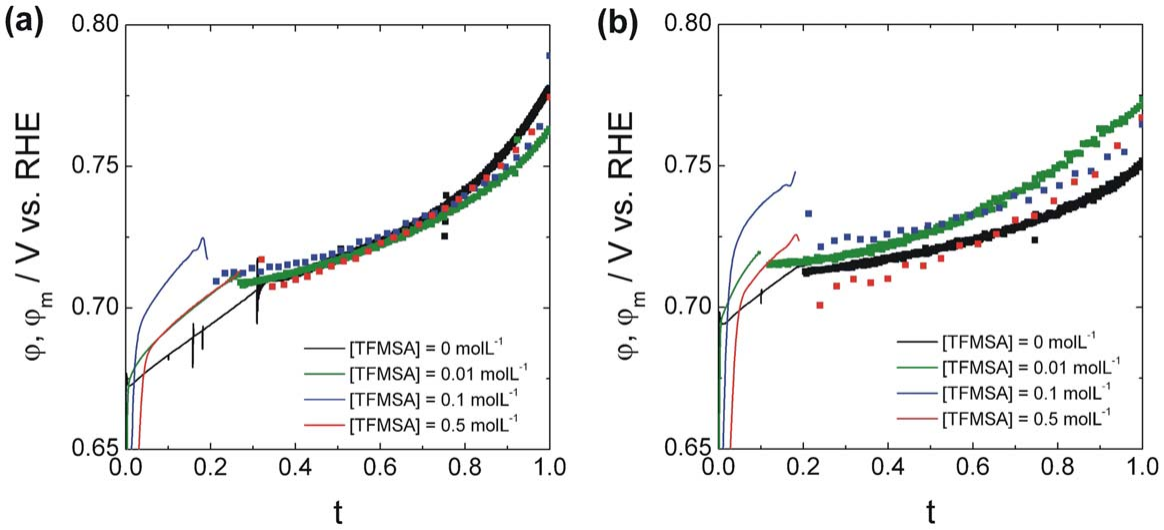
Normalized curves during methanol oscillatory oxidation for selected currents. Galvanostatic curves found for applied currents with values of (a) 0.3 mA and (b) 0.4 mA and different solution compositions. Time was normalized for each series by the instant where 10 oscillations cease to exist.

It is informative to study the evolution of the mean electrode potential, *φ_m_*, during oscillations. Since the ohmic drop found in our experiments was negligible (<2 Ω), the double-layer potential *φ* was assumed to be equal to the measured potential *U,* and *φ_m_* could be computed directly by integrating the potential curve, *φ(t)*, for each oscillatory cycle and dividing the result by the amount of time spent in that cycle:
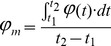



Figure 5 presents the time-evolution of *φ* (along the induction period, continuous line) and *φ_m_*, for two intermediate currents and different TFMSA concentrations. Time in these plots was normalized in terms of the amount of time spent until the harmonic oscillations were finished. Although the curves during the induction period are not superposed, it is seen that oscillations start at about the same range of normalized time, e.g. 20%–40% of the total time. When it comes to the oscillatory region, it is remarkable that all curves fall upon each other quite well. More impressive, however, is that oscillations are initiated and extinguished at about identical values of potential for several different concentrations of TFMSA.

Along the induction period there is an approximate linear increase of the electrode potential. The rate of this increase was found to be inversely proportional to the induction period itself, i.e., those systems whose potential rises faster start oscillating first. For the oscillatory region it can be seen that the curves of average potential display a positive concavity. For now the curves will be fitted by a linear expression so that a net rate of increase of potential can be evaluated. Later on section 4 an explanation for why the transients are not linear will be presented.

**Figure 6:**
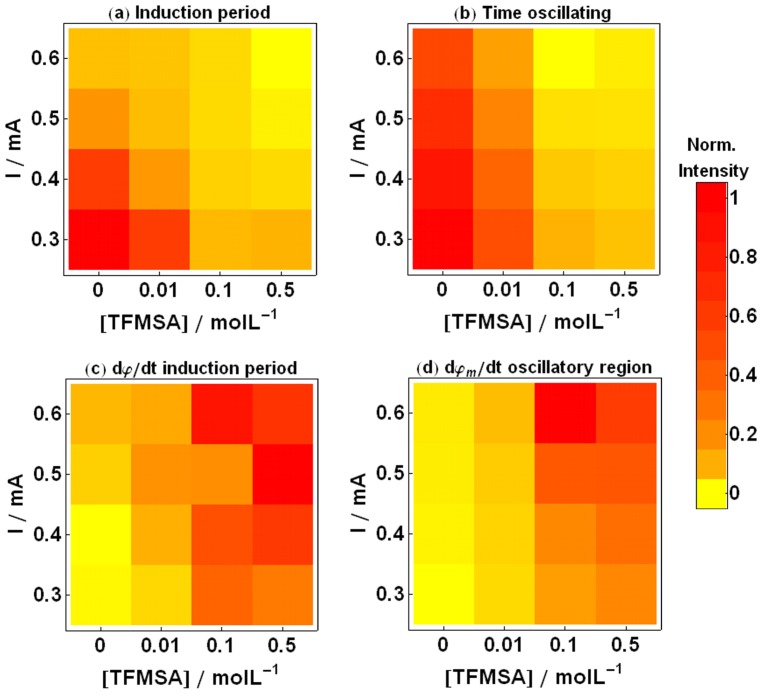
Oscillatory features as a function of the applied current and amount of anion. Maps obtained with data extracted from Erro! Fonte de referência não encontrada. relating the induction period, the time that the system spends in oscillatory region and the rate of change of potential during the induction period for several values of applied current and composition.

Figure 6 shows several maps synthesizing this information. It shows how the induction period, the rate of change of potential during this period and the amount of time the system spends oscillating relate to the applied current and composition in solution. Values were normalized so that the higher one corresponded to 1 (red) and the lower bound to 0 (yellow).

Figure 6 shows that as the amount of anion is increased there is a decrease in how much time it takes for the system to start oscillating and a decrease also on how long it stays in the oscillatory regime. Those trends are followed by an increase in the rate of change of the average potential for both regions. There is a positive correlation between the amount of time spent at the induction period and the amount of time the system oscillates and, also, a negative correlation between those quantities and the rate of change of potential during the induction period.

It is clear from the results presented so far that the oscillatory oxidation of methanol is a transient phenomenon. We have recently used the average value of potential to picture this process and with the aid of spectroscopic results were able to correlate this drift with a diminishment in the amount of adsorbed CO [44]. Nagao *et al.* [45] proposed that the observed transient is the result of two coupled set of processes: a fast one that is responsible for the oscillatory behavior and a slow one that moves the system across a different set of parameters that cannot be completely controlled experimentally. The authors hypothesized that this slow drift was the result of blockage of active sites by oxides and were able to revert the process and stabilize oscillations by applying a gradual decrease in the value of applied current [45].

Since a huge amount of data related to the non-stationarity of methanol oscillations under the influence of TFMSA have been gathered in this work, it is possible to relate those results to the ideas presented in the preceding paragraph. In order to do so, we start with a simple and general view of an oscillating electrochemical system. Under galvanostatic control the applied current (*I*) comprises faradaic (*I_F_*) and capacitive (*I_C_*) contributions,
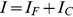
(1)the first one results of electrochemical reactions, whereas the second one accounts for the capacitive charging of the electrical double-layer:



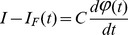
(2)Expression 2 is a differential equation that relates the evolution of potential, *φ*, as a function of the applied current and the kinetics of reaction. In order to understand the effect of the drift it is imperative however to develop an integrated form for the expression. We may start by assuming that the potential of the electrode is a nonstationary oscillatory function of time:
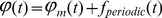
(3)


Where the behavior of *φ* is understood as the sum of a periodic function *f* and an evolving average potential *φ_m_*. One can then apply expression (3) on (2) and integrate over an integer number of oscillatory cycles to obtain^1^:
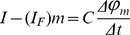
(4)


Expression (4) shows how the average potential depends on its analogue for faradaic current. Since the applied current is constant, the general increase in the rates of evolution of potential during the transient must be either attributed to a decrease in the capacity of the double-layer or to a decrease in the intrinsic activity for methanol oxidation. It is known that the capacity of the double layer generally increases with anion concentration [55] so this factor cannot be responsible for the observed trend. Thus, the increase in the rate of the potential evolution must be caused by a decrease in the activity for methanol oxidation as the amount of anion increases, i.e.,
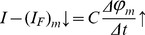
(5)


These conclusions are supported by the voltammetric data displayed in Figure 3, which shows a general decrease in the intensities of organic oxidation as the anion concentration increases. It is also evident in that figure and the discussion that follows that the diminishment in the activity for oxidation is directly related to the blockage of active sites by adsorbed TFMSA. We recall that the faradaic current is directly proportional to the available free sites and obtain:
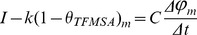
(6)


Where k is the rate constant that depends on the number of electrons exchanged during organic oxidation and the velocity of this process. Finally, the anion effect becomes evident if we analyze the mathematical characteristics of a Langmuir isotherm, [^56^].
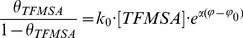
(7)


Here, k_0_ and α are thermodynamic constants dependent upon temperature and the whole expression shows that the ratio between occupied sites against free ones always increases with the increase of the anion concentration or the potential of the electrode. As we have seen, the anion adsorption will lead to a decrease in the values of methanol oxidation, which, in turn, leads to a potential increase. What we can understand now is that higher anion concentration will lead to a faster blockage of the surface (equation 7) and make the average potential change faster (equation 6). Thus, systems with high anion concentration will reach faster the potential at which oscillations become unstable and the oxygen reaction takes place.

The same reasoning may be used to explain the behavior of the induction period. Since no oscillatory behavior is found in that region, there is no reason to use averaged equations. Equation 2 shows that the greater the anion coverage is, the lesser is the value of the faradaic current and consequently the greater is the rate of increase of potential. High concentration of TFMSA implies shorter induction period.

### Oscillation Waveform

Along with changes in the average value of potential during a typical time-series, variations in the pattern of oscillations can also result of the chemical perturbation, as Figure 4 sketches. Simple periodic oscillations may give room to period-2 ones where each cycle repeats after 2 consecutive peaks and through a sequence of period-doubling might reach a situation where mixed-mode oscillations, and eventually chaos, are present. Parameter-plane diagrams can be constructed relating morphology and important features of the experiment such as concentrations and applied currents. Comparable diagrams have been also reported for the case of formic acid, and formaldehyde oxidation under the influence of chloride anions [57], [58].

**Figure 7:**
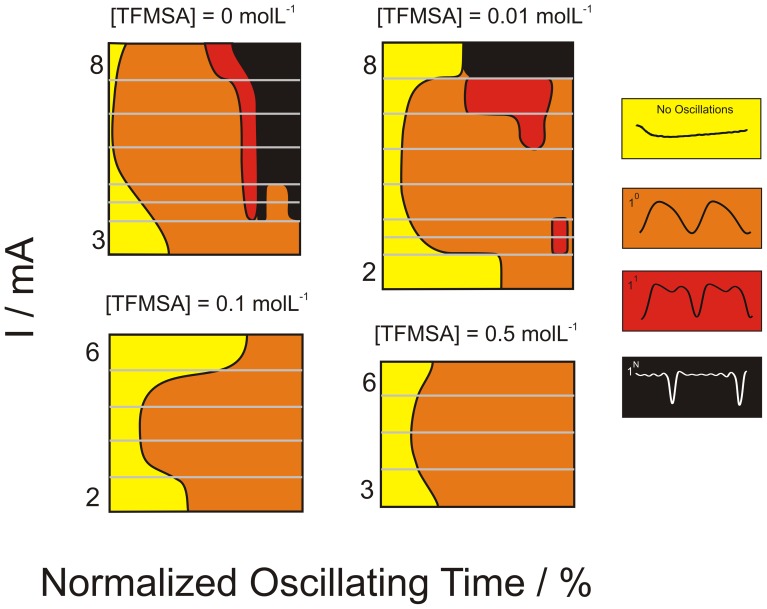
Morphology of oscillations with applied current and amount of anion. Maps presenting the evolution of the morphology of oscillations during the galvanostatic experiments. Each horizontal stripe represents an actual applied current and the gray lines divide different values of current.

Figure 7 shows the evolution of temporal patterns observed during a typical galvanostatic experiment for several compositions of the solution. Oscillations are color-coded according to its morphology (1^0^, 1^1^, or 1^N^) and the stripes delimited by the horizontal white lines account for a given current value used in the galvanostatic experiments. The boundaries between regions were smoothed for clarity of presentation. In these diagrams, time is normalized by the total experiment time, i.e. from the beginning of the experiment to the end of the oscillations. Taking as an example the first diagram with [TFMSA] = 0 and I = 0.8 mA, it is seen that the curve first develops in a stationary fashion and enters the region of simple harmonic (1^0^) oscillations. As time passes the oscillations become less harmonic and eventually change its morphology to period-2 (1^1^). Finally the amount of small amplitude modulations per cycle increases just before the electrode potential enters the oxide region and oscillations are halted. Two major trends can be observed in Figure 7. First, for a given electrolyte composition higher values of applied current resulted in time-series that although short-lived (see Figure 6) displayed a higher percentage of complex oscillations. Second, as the amount of TFMSA in solution increases those complex features gradually become less prominent eventually fading for concentrations higher than 0.1 M.

During a typical experiment, small steady changes occur at the experimental system which are responsible for the evolution of patterns shown in Figure 7 at a given current and composition. It can be noted firstly that as the amount of anion increases the range of values of current which manifest oscillations is decreased along with the maximum value of current that display the phenomenon. As observed when TFMSA is absent, low values of current display mostly simple oscillations explaining in part the features found. As we have seen in Figure 6 an increase in the concentration of TFMSA led to a higher pace of change of the potential of the electrode which suggest that as the system slips faster through the hidden parameter region there is less time for complex oscillations to develop. Those two aspects namely the lower values of applied currents and the faster movement along the oscillatory region can be phenomenologically used to predict the observed trends but should be understood as part of a bigger picture. It is known that for complex oscillations to arise there must exist at least three degrees of freedom. In electrochemical systems, besides the electrode potential and coverages of adsorbed species, the transport of active species to/from the electrode surface might become essential to describe the dynamics and thus provide this additional degree of freedom [16], [17], [59]–[60], [61], [62]. In the present situation, the electrode is kept stationary and mass transport of methanol would limit the surface reaction mainly when the rates of consumption of methanol are high, i.e. when high values of current are applied. Along with that, it takes some time until the dynamics of the oscillator in solution are comparable to the surface one. That discussion explains why low values of current and high values of TFMSA in solution are associated with simpler oscillations. This scenario just discussed is the simplest one able to explain the evolution of patterns as function of the concentration of TFMSA, but it is important to say that there might exist alternative explanations. For example, surface heterogeneity is an important parameter in electrochemistry, particularly for polycrystalline electrodes, and may constitute the second degree of freedom necessary for complex oscillations to arise. In fact, it has been shown that even considerably simple reactions such as that for CO may display complex spatial distribution [63] and that electrochemical oscillatory reactions can possess a component in space [64]. Thus TFMSA could be acting on this parameter inhibiting specific sites by adsorption. Recently the distribution of soluble products was also found to be important for the understanding of oscillatory reactions and the anion could affect, through site blockage, that quantity as well [65].

### Assessing the Sensitiveness

After discussing the mechanistic aspects of the chemical perturbation, namely the addition of different amounts of TFMSA, we have discussed its effect on some features of our electrochemical oscillator. In the following we summarize and discuss the main aspects regarding how electrochemical oscillations may be sensitive towards surface blockage.

It has been shown that, irrespective to the chemical perturbation, during a typical galvanostatic experiment, oscillations may develop and gradually change its properties eventually coming to an end. The primary effect of the chemical perturbation is to shorten the oscillating time. An important result presented in section 3.2 is that the average value of potential increases during experiments of methanol oxidation for both the induction period and the oscillatory region. In fact, the rate of increase was catalogued and found to be a sensitive parameter with respect to the presence of anions in solution, generally increasing as function of its concentration. As a phenomenological manifestation of this drift, it was found that the induction period generally decreases with anion concentration along with the total duration of the oscillatory region and the number of oscillatory cycles.

Starting from a simple and general mathematical expression it was possible to demonstrate that the velocity of this drift was inversely related to the activity of the electrode towards methanol oxidation. Finally the effect of added TFMSA became clear when it was acknowledged that through a gradual blockage of the active sites the anion would decrease the rates of oxidation and consequently enhance the drift. At this point it is convenient however to recall expression 6 and observe that it can be generalized to encompass other types of surface poisoning processes:
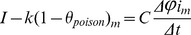
(8)


The important condition in order for expression 8 to hold is that *θ_poison_* increases with potential and is not consumed by any other parallel process. This is the case not only for anion adsorption but for oxide production as well, thus encompassing the hypothesis suggested by Nagao *et. al* [45].

**Figure 8:**
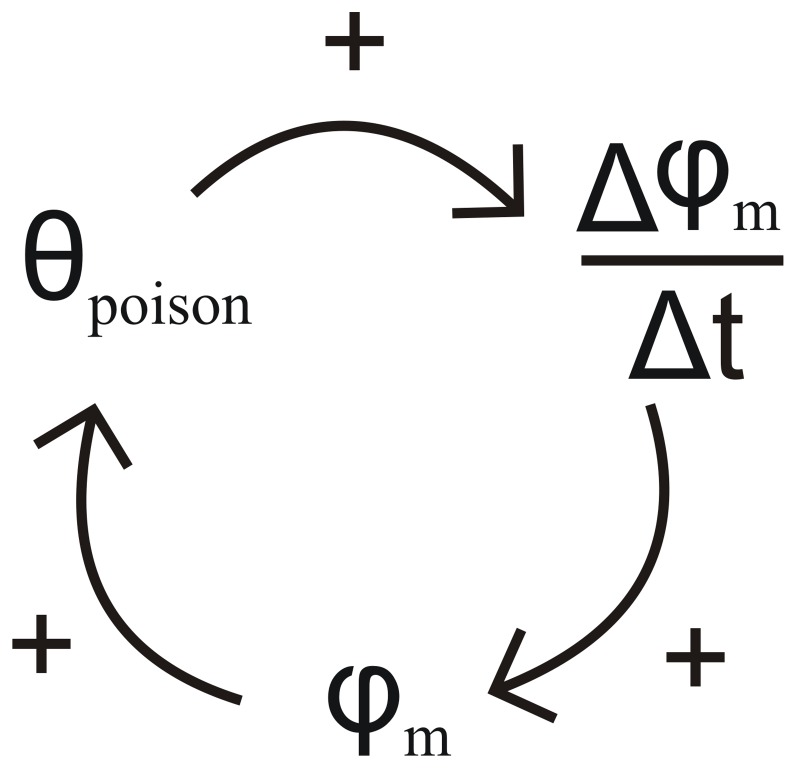
Schematics of the autocatalytic cycle envisioned to be responsible for the instability in oscillations.

Therefore, on top of the coupled feedback loops of the core-oscillator, the unperturbed system can be thought as subjected to a slow drift along the parameter space. The positive feedback loop illustrated in Figure 8 summarizes this process. Starting at a given value of average potential φ_m_, Figure 8 tells that a positive drift (Δφ_m_/Δt >0), causes a further increase in φ_m_. This is a typical process for the negative differential resistance (NDR) region and, generally for the electro-oxidation of small organic molecules, is associated to the surface oxidation. Dissolved anionic species acts as surface poison and its surface concentration increases with the electrode potential, similarly, thus, to that for the formation of oxygenated species. Therefore, concomitantly to the natural drift due to the slow surface oxidation, the poisoning species (TFMSA) introduced as our chemical perturbation reduces auto-catalytically the number of oscillations by reducing the existence region of the core oscillator.

Nonlinear properties and its relationship with the chemical environment have been explored in the literature with success for the determination of specific agents. Gao and coworkers [66], for example, showed that the amplitude of oscillations for the peroxide/thyocianate system was dependent on the amount of an organic in solution. In the field of electrochemistry and generally to surface sciences, adsorbent species may alter drastically the nonlinear behavior as corroborated in this paper. The endurance of oscillatory behavior during methanol oxidation revealed itself to be highly sensitive on the presence of TFMSA in solution as a result of the enhancement of the destabilizing autocatalytic cycle described later. The average amplitude of oscillations also increased, although less dramatically than the former feature. On the other hand, the oscillatory frequency remained nearly unchanged indicating that the core oscillatory mechanism remains barely affected by the perturbation.

## Summary and Conclusions

**Figure 9:**
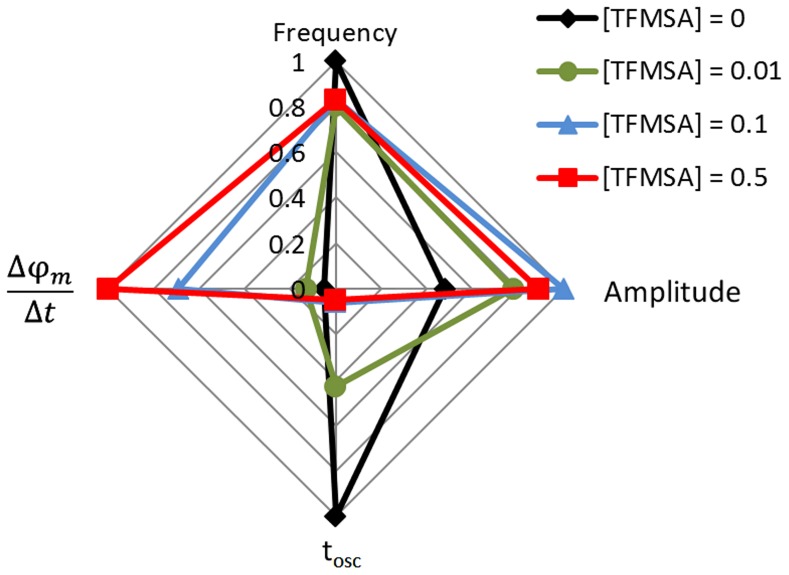
Summary. Radar graph relating the effect of chemical perturbation on the main features of the oscillatory electro-oxidation of methanol on platinum.

We have studied in this paper the effect of chemical perturbation on the dynamics of a typical electrochemical oscillator, namely the electro-oxidation of methanol on platinum. The chemical perturbation was achieved by addition of different amounts of trifluormethanesulfonate (TFMSA). After the initial characterization of the system under non-oscillatory conditions, the effect of chemical perturbations was discussed in terms of the main oscillation parameters, and the sensitiveness of the oscillator assessed. Figure 9 summarizes the main results.

In short, the chemical perturbation affects mainly the duration of oscillations, t_osc_, by accelerating the natural drift already present in the unperturbed system and, thus, anticipating the collision with the fixed point at high potential. As a consequence of the decrease in the oscillatory window, the appearance of more complex waveforms is also prevented as the system is perturbed. In contrast, the oscillatory frequency and amplitude are considerably robust and remains both nearly unaffected by the chemical perturbations. In this way, these two aspects cannot be used to anticipate the oscillation extinction. The co-existence of sensitive and robust properties seems common in complex systems, including biological structures and networks. The dynamic nature of our experimental oscillator, namely the co-existence of two disparate time-scales (i.e. the core oscillator and the slow drift) is also very general and it is arguable that the observed results could be to some extent extrapolated to other less obvious comparable systems.
